# Simultaneous measurement of 16S-rRNA and pre-16S-rRNA as a strategy to monitor clinical tuberculosis

**DOI:** 10.3389/frabi.2026.1760862

**Published:** 2026-04-08

**Authors:** Evelin Dombay, Wilber Sabiiti, Daniela Alferes de Lima Headley, M. Bonifác Légrády, Nina van Campen, Sanne Zweijpfennig, Martin J. Boeree, Derek J. Sloan, Stephen H. Gillespie

**Affiliations:** 1 School of Medicine, University of St Andrews, St Andrews, United Kingdom; 2 School of Chemistry, University of St Andrews, St Andrews, United Kingdom; 3 Department of Pulmonary Diseases, TB Expert Centre, Research Institute for Medical Innovation, Radboud University Medical Center, Radboudumc Center for Infectious Diseases, Nijmegen, Netherlands

**Keywords:** clinical trials, diagnostics, PCR, treatment monitoring, tuberculosis

## Abstract

**Background:**

Culture-based biomarkers of TB treatment response monitoring, e.g., Mycobacterial Growth Indicatory Tube (MGIT), are compromised when bacteria enter a non-replicating persister phase limiting the measurement of antibiotic efficacy and resistance. Understanding how antibiotic exposure to antibiotics alters bacterial physiology could help develop more effective TB therapies. We developed a novel assay with simultaneous measurement of 16S rRNA (bacterial burden) and its precursor, pre-16S rRNA (metabolic activity), and tested it on samples from patients in a trial of optimised-dose rifampicin.

**Methods:**

We developed a multiplex reverse transcriptase quantitative PCR assay (RT-qPCR) to measure relative gene expression of pre-16S rRNA and 16S rRNA in pre-treatment (control) and sequential samples from patients in the Phase II HIGHRIF2 (NCT00760149) clinical trial. We constructed a mathematical model to assess changes in pre-16S gene expression relative to 16S rRNA over time, facilitating the comparison of rifampicin doses’ efficacy.

**Findings:**

In a retrospective study of 19 patients, pre-16S rRNA and 16S rRNA decreased steadily during the initial 36 days of treatment. This was evidenced by the rising cycle threshold (Cq) values slope 0.404 and 0.212, respectively, however, pre-16S rRNA decreased significantly quicker (P<0.0001). The changes in the relative gene expression of pre-16S rRNA during treatment fitted a double exponential decay curve (R^2^ = 0.996). According to this model, 1200 mg RIF-containing therapy exerted the most potent and rapid impact on pre-16S rRNA expression (Maximum suppression (R_min)_=1.694, T (time) =9.78 days), and also resulted in the swiftest daily reduction in bacterial load (–0.072 log_10_ CFU ml^-1^/day).

**Interpretation:**

The pre-16S rRNA and 16S rRNA gene expression multiplex PCR reported here provides an easy to use and rapid marker of drug efficacy and has potential to assess the efficacy of existing or novel drug combinations.

## 1 Introduction

Tuberculosis (TB), the world’s leading infectious disease killer, claims approximately 4000 lives each day. While progress has been made, further improvements to combat tuberculosis are essential to further reduce its prevalence and impact globally. However, controlling the spread of the infection is hindered by the lack of effective therapy and tools to measure treatment efficacy in clinical trials (Gillespie et al., 2024).

Monitoring tuberculosis treatment is difficult with target product profiles for immunology and microbiology based approaches being recently published (Gupta-Wright et al., 2023; Gillespie et al., 2024; diNardo et al., 2025). This paper focuses on microbiological end points that are often complicated by the diversity of *Mycobacterium tuberculosis* (Mtb) cell states that are found during infection (Hammond et al., 2015; Lipworth et al., 2016). Moreover, Mtb cells vary in their response to antituberculosis therapy including phenotypic resistance (Negi and Sharma, 2024). Models describing dormancy states and bacterial population dynamics have been developed that can be used to explain the action of TB drugs (Wayne and Hayes, 1996; Betts et al., 2002; Cunningham et al., 2004; Shleeva et al., 2011). Mitchison et al., proposed the existence of four distinct cell types each of which responds to antibiotics differently (Mitchison, 1979). A hypothesis proposes that bacteria can exist in various states including active replication, dormancy, or a transitional phase, characterised by different metabolic activity and gene expression (Shleeva et al., 2002). Cells can transition from dormancy to fully active growth states rapidly. Another theory suggests that dormant, non-replicating bacteria may periodically awaken under favourable growth conditions, acting as “scout” cells that signal other dormant cells to reactivate, potentially leading to a relapse of infection in the host (Epstein, 2009). The importance of this phenomenon is that the non-replicating persister states exhibit increased tolerance to antibiotics, reduced culturability in growth media, and accumulation of intracellular lipids (Raghunandanan et al., 2019). Hammond et al. found that these lipid-loaded dormant cells exhibited a minimum inhibitory concentration (MIC) forty times higher than the lipid-poor phenotype (Hammond et al., 2015). The co-existence of different Mtb phenotypes during infection is further evidenced by the dynamics of bacillary elimination by TB drugs. This process is typically biphasic, involving a early bactericidal phase, where actively replicating bacteria are eliminated within the first 5–7 days, followed by a sterilising phase during which the persistent bacterial population is killed at a slower rate by the “sterilising drugs” rifampicin and pyrazinamide. Mycobacterial Growth Indicator Tube (MGIT) is recognised by the Food and Drugs Administration (FDA) as a biomarker for use in drug development. Many trials use time to MGIT culture positivity (TTP) to measure treatment response, but this can be compromised by overgrowth of other non-TB organisms.

While conventional methods like culture or advanced techniques such as the Tuberculosis Molecular Bacterial Load Assay (TB-MBLA) (Sabiiti et al., 2020a) can assess the early bactericidal activity (EBA) of TB drugs, measuring their efficacy beyond bactericidal potency presents challenges. However, drugs such as rifampicin, known for their action against persistent bacteria, play a significant role in disease control. These antibiotics work by reducing bacterial viability through inhibition of fundamental biosynthetic pathways essential for microbial survival. Consequently, they retain some efficacy even in non-replicating persister with low metabolic activity. Rifampicin and pyrazinamide, the two most potent sterilising agents in the current 6-month therapy, block DNA-dependent RNA synthesis and CoA synthesis, respectively. 16S rRNA serves as a reliable marker of bacterial burden in samples (Desjardin et al., 1999; Sabiiti et al., 2020a). As cells require a stable pool of rRNA for survival, a substantial number of copies persist within the cell, whether actively replicating or not. Moreover, 16S rRNA, does not persist for the same duration as DNA which means that measuring 16S rRNA enables estimation of live organism concentration accurately (Musisi et al., 2024). Cangelosi et al. demonstrated that pre-16S rRNA was present at low concentration in stationary phase cultures but rapidly replenished when bacteria are transferred to fresh media (Weigel et al., 2013). Similarly, research indicates low detectability of pre-16S rRNA in Mtb during low metabolic non-replicating phase (Cangelosi and Meschke, 2014). Beyond reflecting bacterial physiology, pre-16S rRNA serves as a potential marker for phenotypic resistance to antibiotics like rifampicin. This was evidenced in studies using rifampicin-sensitive and resistant *E. coli* strains, as well as investigations into the impact of rifampicin and ciprofloxacin on pre-16S rRNA abundance in susceptible and resistant Mtb strains, which could distinguish between them after 48 hours of antibiotic exposure (Cangelosi and Brabant, 1997; Cangelosi and Meschke, 2014).

Building upon these insights, we developed a multiplex RT-qPCR-based assay for simultaneous measurement of 16S rRNA and pre-16S rRNA levels applicable to *in vitro* and clinical sputum samples from patients in a Phase 2b clinical trial (Aarnoutse et al., 2017). Our aim was to illustrate the metabolic heterogeneity of Mtb during infection and evaluate the impact of sterilising drugs on distinct pathogen populations.

## 2 Methods

### 2.1 *In vitro* assessments

To investigate the concentration changes of different markers during *in vitro* growth, *M. tuberculosis* H37Rv cultures were grown in Middlebrook 7H9 with 10% ADC broth and sampled on days 2, 5, 16, 20, and 35. At each time point, 1 ml of culture was removed, RNA was extracted, and 16S rRNA and pre-16S rRNA levels were measured using the multiplex RT-qPCR assay (VitalBacteria™ UK) (Sabiiti et al., 2020b). Bacterial load was calculated using a previously established 16S rRNA vs. CFU/ml standard curve.

### 2.2 Clinical samples for evaluation

We conducted a retrospective study involving 19 randomly selected patients from the HIGHRIF2 (NCT00760149) clinical trial receiving a fixed dose isoniazid, pyrazinamide and ethambutol with supplemented rifampicin to make a final dose of 600 mg, 900 mg, or 1200 mg (Aarnoutse et al., 2017). Sputum from the first five weeks of therapy was preserved (Oragene, RNA) and only participants with a complete set of samples were included in the study.

### 2.3 RNA extraction and reverse-transcriptase quantitative real-time PCR

For assay development and evaluation, RNA extraction and DNase treatment followed by the TB-MBLA protocol (VitalBacteria™), as detailed in the **Supplementary Material** (Gillespie et al., 2017). Briefly, thawed samples were homogenised with N-acetyl-l-cysteine (NALC) solution, vortexed, and aliquoted. An internal control was added, and the mixture was centrifuged at 20,000x g for 10 minutes. Initial RNA extraction steps were carried out in a BSL3 laboratory until safe handling in a BSL2 environment was feasible. RT-qPCR was performed on a RotorGene 5plex platform (Qiagen) in the BSL2 laboratory. Primers and probes for *M. tuberculosis* 16S rRNA and the internal control (IC) (Eurofins Genomics, Germany). To establish a standard curve, known CFU/ml extracts of 16S rRNA were amplified. RNA extracts from patient samples were diluted 1:10 to prevent PCR inhibition during quantification. Raw PCR data were used to estimate bacterial load, adjusted by multiplying the log_10_ CFU/ml by 10 (Sabiiti et al., 2020a).

### 2.4 Pre-16S rRNA/16S rRNA ratio analysis

Pre-16S/16S rRNA gene expression ratio analysis was undertaken by using the Livak delta-delta Ct method (Livak and Schmittgen, 2001). To determine the relative difference in the expression level during treatment, we normalised the Ct of the target gene (pre-16S rRNA) to the reference (16S rRNA, housekeeping gene) gene for both the test sample (sample taken during treatment) and the calibrator sample (sample taken prior to treatment) (Δ*Ct*). We normalised the delta Ct of our test sample to the calibrator sample (ΔΔ*Ct*). Finally the fold change in relative gene expression was calculated by using 2^–ΔΔ^*^Ct^* formula. Data for each of the patients were calculated independently.

### 2.5 Mathematical modelling

**Figure 1:**
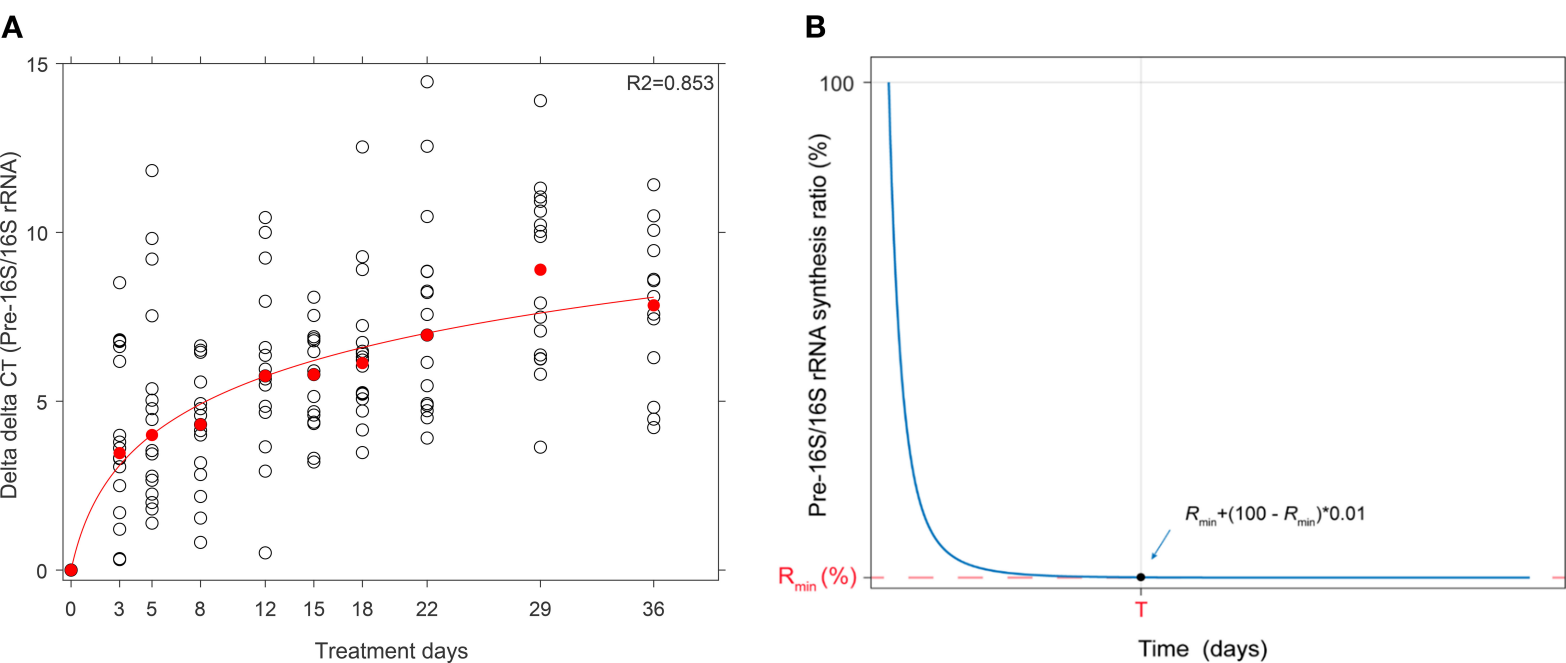
**(A)** Graphical representation of the time dependence of AACt by an exponential saturation curve fitted on median ▵▵Ct values (red) measured in a cohort of 19 patients. The rising trend in ▵▵Ct indicates a depleting concentration of pre-16S rRNA in during treatment vs. pre-treatment. **(B)** Mathematical expression of the pre-16S rRNA expression fold change described by a double exponential decay curve. The two variables, Rmin and T, represents the maximum suppression percentage and the time when 99% of the Rmin is reached, respectively.

In order to find a parametric form that describes the changing ratios over time, we formulated a mathematical model and fitted a double exponential decay curve to the changing gene expression profile. The mathematical model was developed using MATLAB R2019a software (The MathWorks, Inc, 2019). First, the time dependence of ΔΔCt had to be expressed since the fold change in pre-16S rRNA expression was calculated by raising 2 to the power of –ΔΔCt Equation Equation 1. If the function were linearly increasing, the expression ratio would have been represented by a monoexponential decay. In this case we found that the expression ratio was best described by an exponential saturation curve as shown in **Figure 1A**. A saturation curve starting from 0 and approaching a maximum value has the general form of Equation 2. Inserting Equation 2 into Equation 1 showed that the time dependence of the expression ratio of genes followed a double exponential decay, as given in Equation 3.

(1) $$R(t)=2^{-\Delta \Delta Ct(t)}$$

(2) $$\Delta \Delta Ct(t)=\Delta \Delta Ct_{\max}(1-e^{-At})$$

(3) $$R(t)=2^{-\Delta \Delta Ct_{\max}(1-e^{-At})}=2^{-\Delta \Delta Ct_{\max}}\cdot 2^{\Delta \Delta Ct_{\max}\;e^{-At}}$$

In Equation 3 the $2^{-\Delta \Delta Ct_{\max}}$ prefactor corresponds to the minimum asymptotic value that the function is approaching and, therefore, this is denoted by $R_{\min}$, which led to a simple formula for the time dependence of the expression ratio.

(4) $$R(t)=2^{-\Delta \Delta Ct_{\max}(1-e^{-At})}=R_{\min}^{\left(1-e^{-At}\right)}$$

In Equation 4*A* is the exponential rate constant, which determines the rate of decay, however, this parameter did not provide direct insight into how quickly *R*_min_ is approached. We recast Equation 4 and used a *T* parameter instead, which corresponds to the time point when 99% of the total decrease is reached. Although this parametrisation somewhat complicated the mathematical expression (Equation 5), gave a more natural interpretation of the parameter in the equation.

(5) $$R(t)=R_{\min}^{1-{\left(\frac{\ln(100R_{\min})-\ln(1+99R_{\min})}{\ln(R_{\min})}\right)}^{\frac{1}{T}t}}$$

Curves corresponding to Equation 5 were fitted to experimental data with the only difference that percentages are used instead of fractions. The meaning of the parameters Rmin and T are illustrated in **Figure 1B**.

The changing dynamic in ΔCt values suggesting a rapid reduction in pre-16S rRNA expression during *in vivo* drug exposure. In relative gene expression RT-qPCR ΔCt is the difference between the gene of interest (pre-16S rRNA) and the reference gene (16S rRNA) which in this case is also used as the marker of bacterial burden. Therefore, the quantitative dependence of pre-16S rRNA on the total burden of bacteria can be measured.

### 2.6 Data selection and statistical analysis

To verify the presence of low abundant targets in the high Cq samples, a high-resolution melt-curve analysis was performed. RNA extract with a pre-16S rRNA Cq value of 37 produced a well distinguishable peak at 80-85 °C without any additional peaks from non-specific amplification and/or primer-dimers (see **Supplementary Material**). Thus, for the absolute quantification of bacterial loads, Cq ≤ 30 was considered, whereas for the gene expression analysis, Cq<40 was included in the analyses. Only samples that showed no amplification in negative controls (water and minus Reverse Transcription [-RT] control) were included in the analyses. Negative samples (no amplification detected) were assigned a Cq value of 40 where appropriate.

Statistical analyses were performed using GraphPad Prism 8.0.1. Mean and median values were calculated using standard formulae. Cq values converted into estimated CFU/ml (bacterial load) were log transformed prior to data analysis. Statistical differences between treatment groups were assessed using one-way analysis of variance (ANOVA) or the Kruskal-Wallis test. F statistics were used to compare the rate of sputum bacillary reduction across treatment arms. Correlations between different biomarkers were evaluated using Spearman’s rank correlation test. Multiple linear regression analysis was conducted to analyse the relationship between MGIT TTP (time to positivity) versus bacterial load and the fold change in pre-16S rRNA gene expression. A probability (p-value) less than 0.05 was considered statistically significant.

## 3 Results

### 3.1 Changes in the level of Mtb-specific molecular markers during treatment

**Figure 2:**
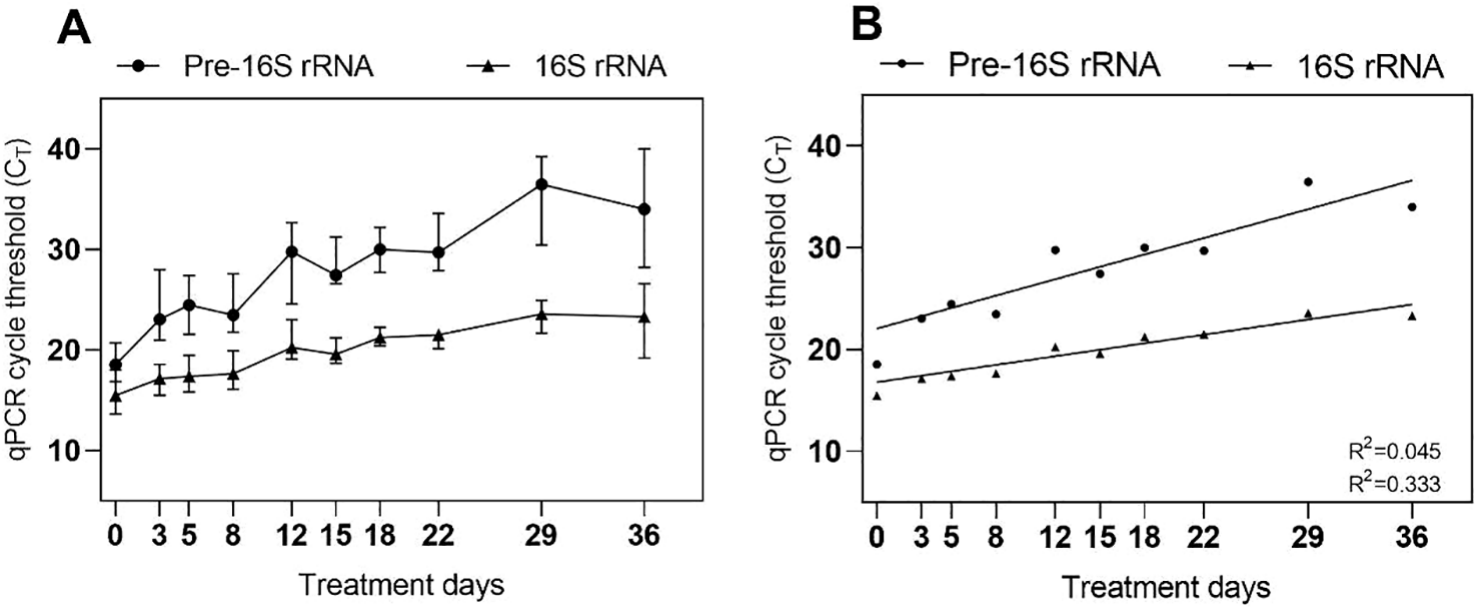
Raw RT-qPCR Cq values before **(A)** and after linear regression analysis **(B)**. The increasing Cq values reflect a depletion in both pre-16S rRNA and 16S rRNA due to their inverse correlation with amplicon concentration. The decrease in 16S rRNA abundance mirrors a reduction in bacterial load, which does not entirely correspond with the decline in pre-16S rRNA production, indicating that the measurable pre-16S rRNA concentration changes independently of bacterial burden. The plots depict median CT values for each patient visit, with error bars representing the range of the data.

Changes in the levels of Mtb-specific molecular markers during treatment are depicted in **Figures 2A, B**, where linear regressions were fitted to Cq values of pre-16S rRNA and 16S rRNA from 190 samples collected within the first 36 days of treatment to assess molecular abundance changes. The positive slopes of the regression lines (0.040 ± 0.035 for pre-16S rRNA and 0.212 ± 0.023 for 16S rRNA) indicate a gradual decrease in the concentration of both molecules, with pre-16S rRNA showing a significantly faster reduction than 16S rRNA (p<0.0001). By the end of the assessment period, none of the patients had negative 16S rRNA results, whereas pre-16S rRNA could no longer be detected in 6 (31.6%) patients.

### 3.2 Suppression of pre-16S rRNA gene expression during treatment

**Figure 3:**
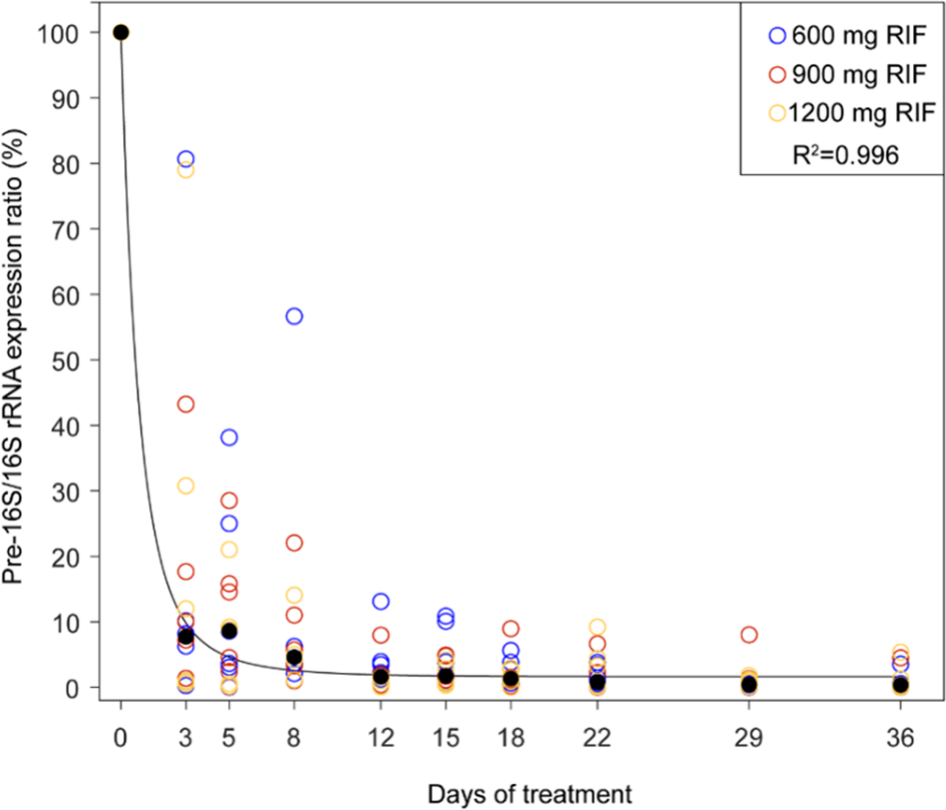
Double exponential decay curve fitted on median pre-16S rRNA fold change during treatment. Each treatment arm is represented by a distinct colour (HR600ZE - blue, HR900ZE - red, HR1200ZE - orange).

The effect of TB therapy, irrespective of rifampicin doses, on the pre-16S rRNA expression levels was demonstrated by fitting a double exponential decay curve on 163 serial samples. The accuracy of the model was confirmed by fitting the curve on the median expression fold change data for each time point yielding a R^2^ value of 0.996 (See **Figure 3**).

### 3.3 Assessment of rifampicin dose-dependent response in bacterial RNA synthesis

We conducted an analysis of pre-16S rRNA gene expression rates during the initial 5 weeks of therapy, examining each treatment cohort separately. The analysis included 7 patients in the 600 mg RIF arm and 6 patients each in the 900 mg and 1200 mg RIF groups. In all instances, there was a notable decrease in pre-16S rRNA expression levels in response to drug exposure. Additionally, we observed a trend in the maximum suppression (Rmin) and the rate of change (T) that corresponded with the increasing doses of rifampicin.

**Figure 4:**
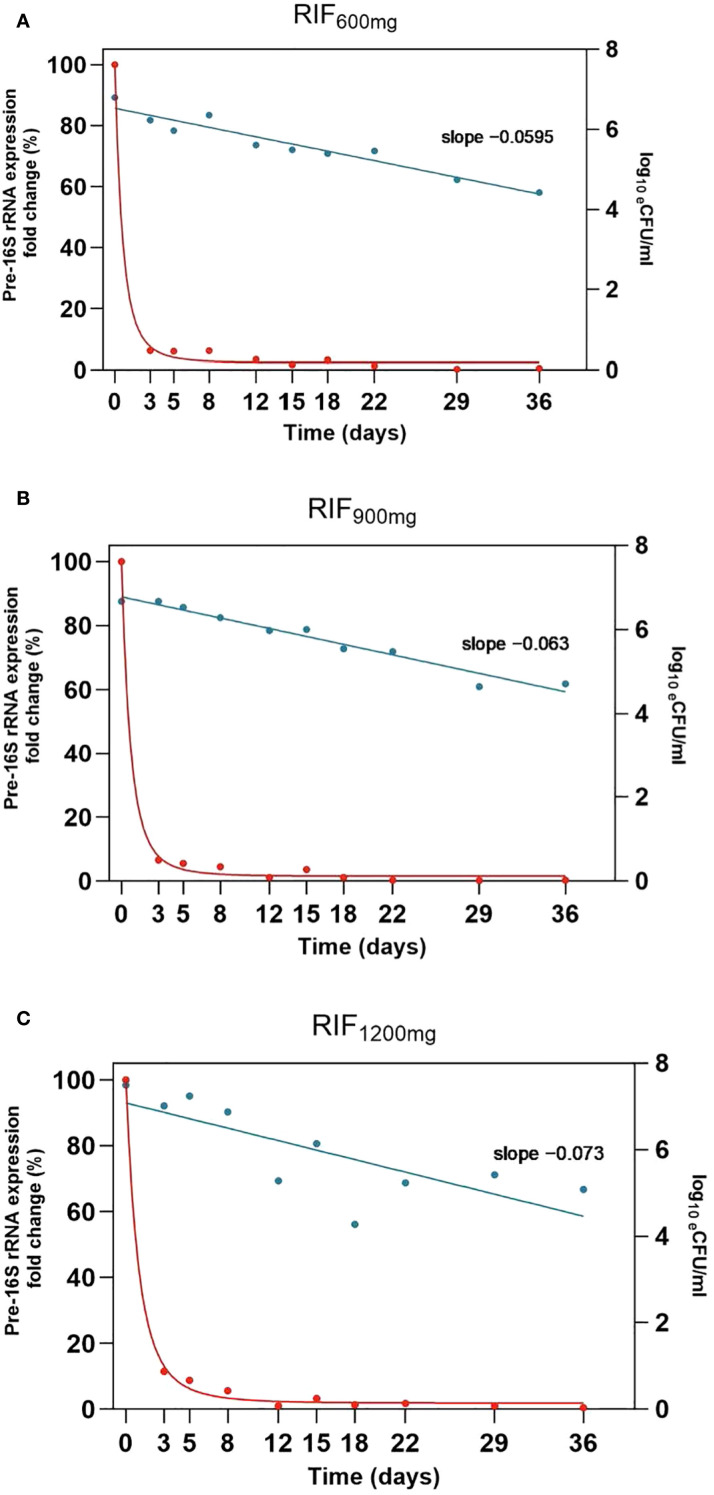
**(a–c)** Plot of pre-16S rRNA expression with plot of Rm in red and the bacteridical activity in green.

**Figure 5:**
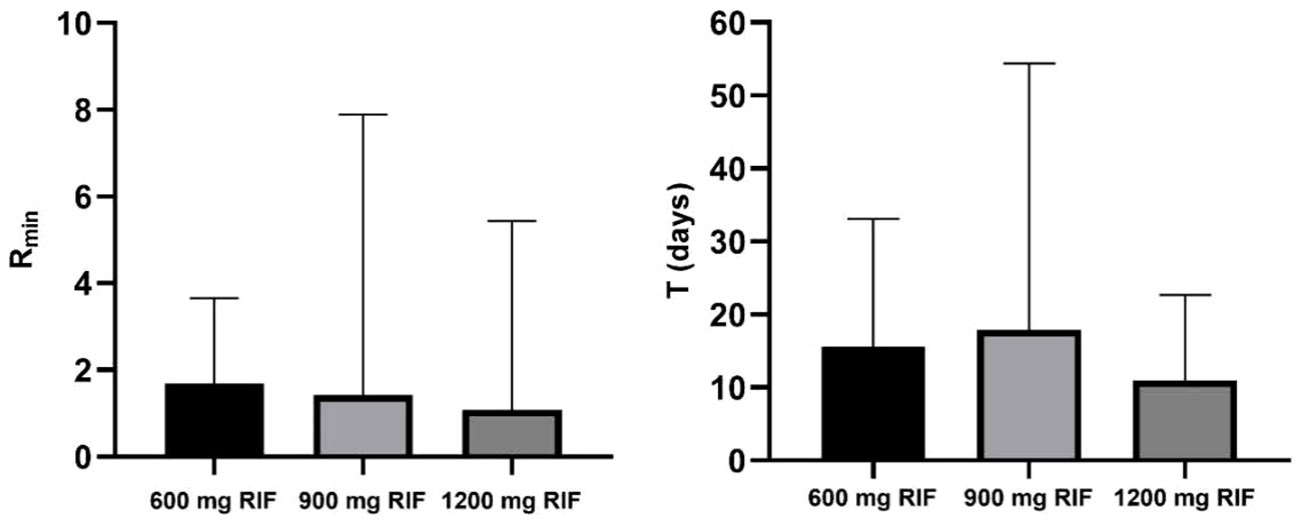
Comparison of maximum suppression rate (Rmin) and time to achieve 99% of Rmin (T) illustrating trends across treatment arms. Columns represent median values with error bars indicating the range of medians. Rifampicin shows a dose-dependent effect on both Rmin and T, with 1200 mg RIF demonstrating the most efficient suppression of de novo ribosome synthesis within a shorter period. Bactericidal activity in decreasing order: RIF1200HZE (-0.072 log10 estimated CFU/mL), RIF900HZE (-0.061 log10 estimated CFU/mL), RIF600HZE (-0.058 log10 estimated CFU/mL) p=0.663. Bacteriostatic activity in decreasing order: RIF1200HZE (Rmin=1.080,T=9.789), RIF900HZE (Rmin=1.424, T=17.87), RIF600HZE (Rmin=1.694, T=12.95) p=0.915 and 0.4698 for Rmin and T, respectively between cohorts.

**Figures 4A–C** and **Table 1** illustrate that with 1200 mg of rifampicin, pre-16S rRNA expression was suppressed by 98.92% within a median (range) of 9.79 (0.534 to 25.57) days of therapy, retaining only a median (range) of 1.080% (0 to 5.430) of the original pre-16S rRNA expression level measured before treatment. The dose-dependency of pre-16S rRNA expression was similarly observed in the 900 mg and standard 600 mg rifampicin-containing cohorts. In the 900 mg group, Rmin was a median (range) of 1.424 (0.012 to 7.885), corresponding to a 98.58% decrease, while in the 600 mg group, Rmin was 1.694 (0 to 3.653), indicating a 98.31% reduction (**Figure 5**). With regards to the rate of change, 1200 mg dose resulted in the fastest suppression, followed by 600 mg and then 900 mg (9.789 [0.534 to 25.57], 12.95 [0.512 to 51.62] and 17.87 [0.758 to 54.41], respectively). A comparative assessment of the changes in bacterial load and pre-16S rRNA gene expression was performed in the three different arms to observe the elimination vs. suppression effect of treatment. **Figure 5** illustrates treatment with 1200 mg rifampicin resulted in the highest daily reduction in bacterial load (–0.073 ± 0.021 CFU ml^-1^/day), followed by 900 mg (–0.06 ± 0.004) CFU ml^-1^/day), and 600 mg (–0.059 ± 0.006 CFU ml^-1^/day). The data also indicate that the gradual decline in bacterial loads, as quantified by 16S rRNA follows a rapid suppression in pre-16S rRNA gene expression within the initial 3 days of therapy. Despite the observed differences in trajectories, none of these reached statistical significance (differences in bacterial load: p=0.663, R_min_: p=0.915, and T: p=0.469).

**Table 1:** Comparison of R_min_ and time to 99% R_min_ (T) in the HR_600_ZE, HR_900_ZE and HR_1200_ZE treatment groups.

Doses of RIF	R_min_ (median, range)	Time (*T*, days)	Goodness of fit (R^2^)
600 mg	1.694 (0 to 3.653)	12.95 (0.512 to 51.62)	0.998
900 mg	1.424 (0.0122 to 7.885)	17.87 (0.758 to 54.41)	0.996
1200 mg	1.080 (0 to 5.430)	9.789 (0.534 to 25.57)	0.996

The number of patients included was 7 in the 600 mg arm and 6 in both 900 mg and 1200 mg arms. R2 was measured by fitting the double-exponential decay curve on median values.

**Figure 6:**
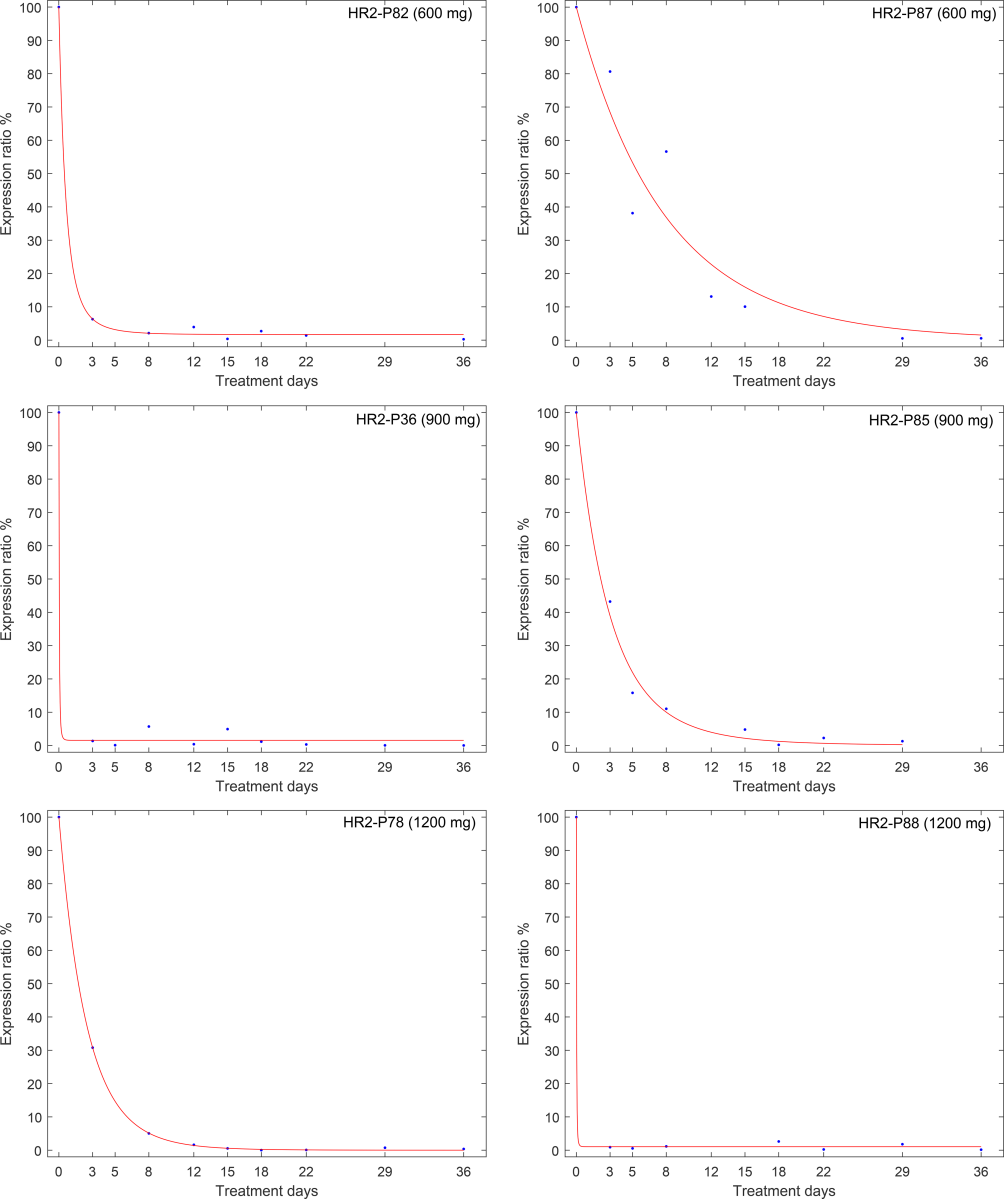
Examples of the diversity of treatment responses depicted by changes in pre-16S rRNA expression during treatment, measured in a representative sample of 6 patients from the HIGHRIF 2 clinical trial.

Our analysis also highlighted significant variability among patients in the rate of pre-16S gene expression suppression. While some patients exhibited maximal suppression within a few days, other patients showed a slower and less pronounced response (**Figure 6**).

### 3.4 Pre-16S rRNA gene expression during *in vitro* growth vs. drug treatment

To provide a bacteriological interpretation of early-stage low pre-16S rRNA gene expression during treatment, we conducted an *in vitro* time-to-suppression experiment using the Mtb H37Rv strain. This allowed us to track changes in ribosome synthesis profiles across well-characterised *in vitro* growth stages, providing a reference for observations made in the clinical cohort.

**Figure 7:**
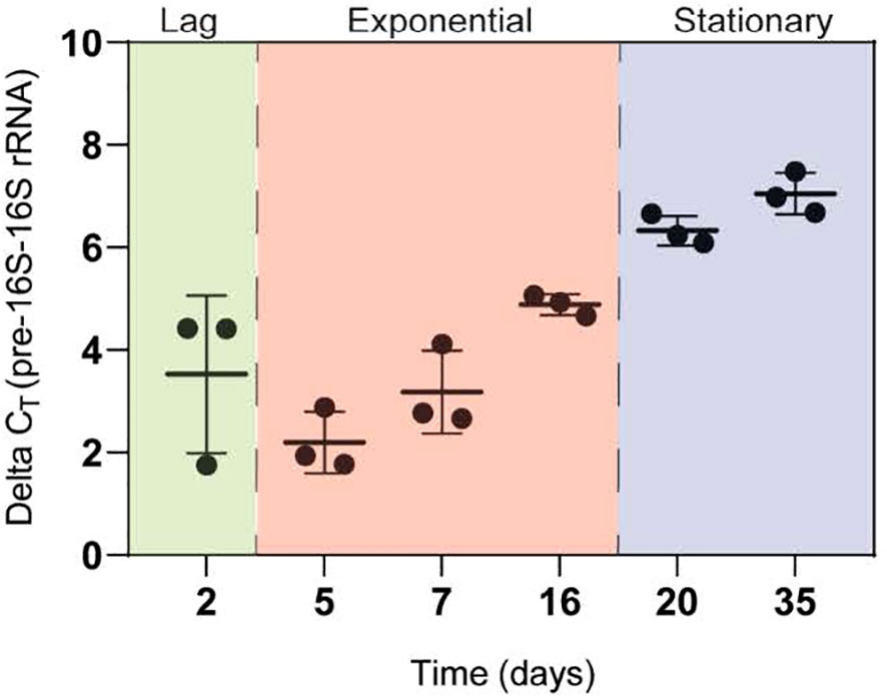
▵CT measured at different stages of in vitro growth. The progression from active replication in the logarithmic phase to the metabolically less active stationary phase is demonstrated by increasing delta CT values thorughout growth. Determination of the putative phases of replication is informed by the literature and the growth curve previously established using Mtb 16S rRNA.

Initially, we assessed the concentration dependency of pre-16S rRNA relative to 16S rRNA and thus total bacterial burden through a relative expression ratio. This ratio, represented by ΔCt, reflects differences in expression levels between the two molecules. If pre-16S rRNA levels were solely dependent on bacilli count in a sample, ΔCt would remain constant throughout growth phases. However, we observed a consistent increase in ΔCt from the logarithmic to the stationary growth phase. Median ΔCt was 2.77 during the logarithmic phase, indicating pre-16S rRNA levels were similar to 16S rRNA. In contrast, ΔCt rose to 6.61 in the stationary phase, suggesting pre-16S rRNA production had halted, resulting in an approximate 2-log difference between pre-16S rRNA and its mature form, 16S rRNA (**Figure 7**; **Supplementary Table 1**).

In a subsequent analysis, an exponential saturation function similar to Equation 2 but using delta C_T_ data instead of delta-delta C_T_ (Equation 6) was fitted to both the *in vitro* and the clinical dataset.

(6) $$\Delta CT(t)=(\Delta CT_{\max}-\Delta CT_{0})(1-e^{-A\;t})+\Delta CT_{0}$$

**Figure 8:**
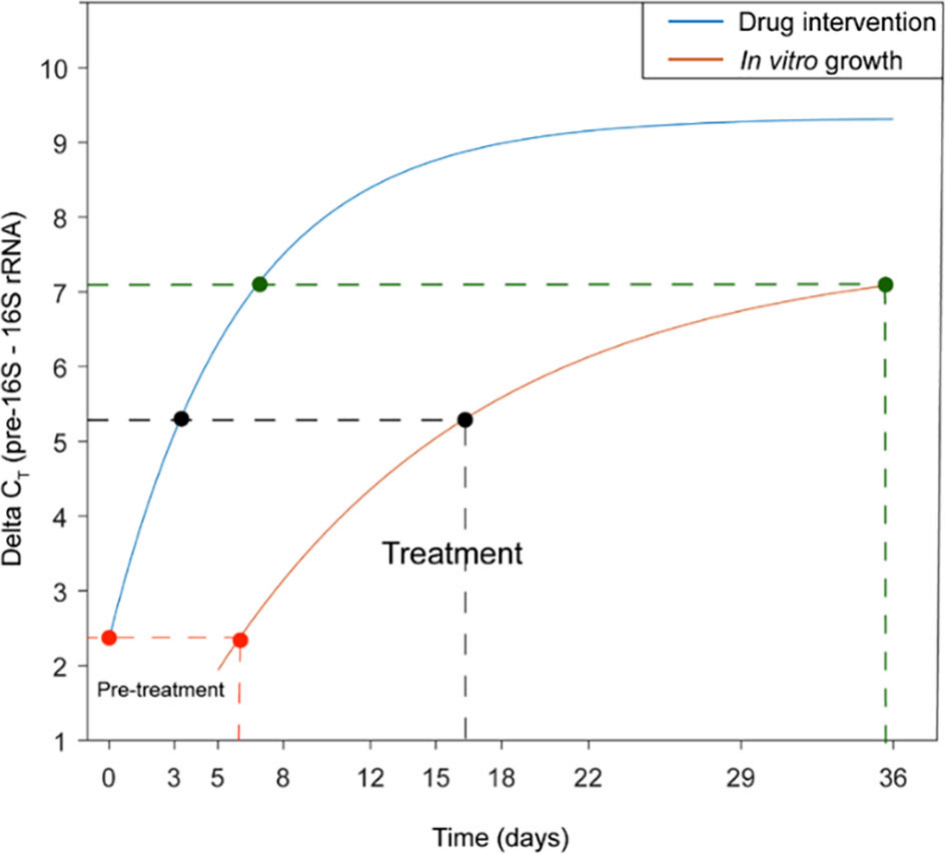
Comparison of the impact of drug intervention observed in clinical samples (blue curve) versus the natural cessation of basic metabolism in in vitro cultures (red curve). The graph illustrates that drug intervention induces a faster metabolic shutdown in bacteria compared to the natural course of microbial growth. Prior to treatment, the samples predominantly consist of actively replicating bacteria. Upon antibiotic treatment, the phenotypic composition rapidly shifts, with a majority of cells entering a state characterized by intermittent metabolic activity, akin to TB bacilli in stationary phase in vitro cultures.

Using this model, we compared the rate of pre-16S rRNA gene expression suppression measured during therapy with that observed *in vitro* and consequently, allowing us to predict the bacterial replication state at different clinical time points. Prior to treatment, the bacterial population resembled growth in the logarithmic phase of a 5-6-day-old bacterial culture. However, after only 3 days of treatment, the ΔCt closely mirrored that observed in an early stationary phase culture aged 16–17 days. By the 6th day of therapy, ΔCt had increased to levels comparable to those observed in a late stationary phase culture aged 35 days (**Figure 8**).

### 3.5 Investigating the relationship between MGIT time-to-positivity and bacterial metabolic activity

**Figure 9:**
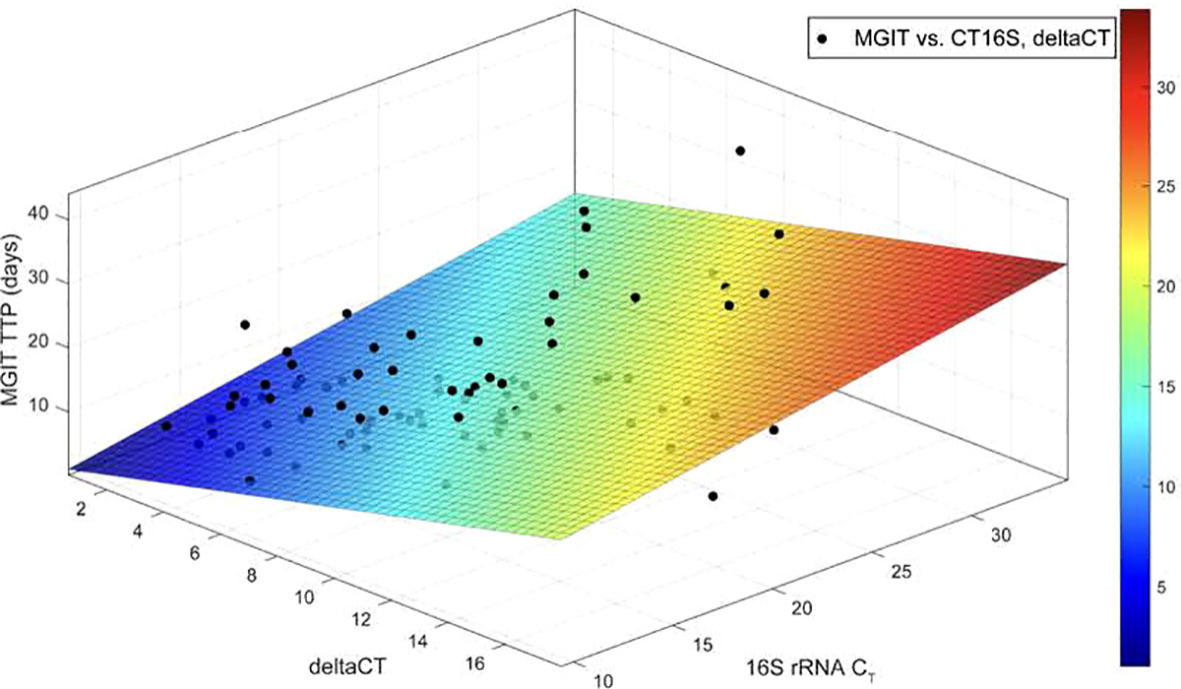
illustrates the multiple regression analysis and the fitted two-dimensional plane showing the relationship between MGIT TTP, ▵CT, and 16S rRNA CT. It displays data points of MGIT TTP against ▵CT and 16S rRNA CT measurements from 19 patients in the HIGRIF2 clinical trial. The colour bar indicates the MGIT TTP values predicted by the model.

In the final part of our study we employed multiplex RT-qPCR and multiple linear regression analysis, to assess the dependency of MGIT TTP on bacterial metabolic activity and burden. The variables measured included MGIT TTP as the dependent variable, ΔCT (pre-16S-16S) as a marker of metabolic activity, and CT 16S as a marker of bacterial burden (inversely proportional; higher CT indicates lower bacterial load). Our hypothesis posited that higher ΔCT values in sputum samples would correspond to longer TTP, while lower CT differences would result in shorter TTP. Employing multiple linear regression, we simultaneously examined the influence of these two independent variables on MGIT TTP. The results of the multiple linear regression analysis are illustrated in **Figure 9** and summarized in **Supplementary Table 2**: The positive slopes indicate a significant association between MGIT TTP and both ΔCT and 16S rRNA CT, with statistically significant interactions observed. An increase of one unit in ΔCT (indicating a change in metabolic state) was associated with approximately a one-day increase in MGIT TTP. Conversely, an increase of one unit in 16S rRNA CT (indicating decreased bacterial burden) was associated with approximately a 0.5-day increase in MGIT TTP. Notably, the association with ΔCT appeared three times more significant than that with 16S rRNA CT, suggesting that MGIT TTP is more influenced by the physiological and metabolic properties of *M. tuberculosis* than by the absolute number of bacteria.

## 4 Discussion

Monitoring the decline in viable count using the tuberculosis molecular bacterial load assay is now well established in research studies and is increasingly being applied in clinical trials (Mbelele et al., 2021; Musisi et al., 2024). The correlation between the reduction in 16S rRNA concentration and viable counts, traditionally measured by colony counts or time to positivity in Mycobacterial Growth Indicator Tube (MGIT) (Gillespie et al., 2014), represents a significant advance in treatment monitoring as it is more rapid and, importantly, not subject to bacterial contamination that causes the loss of samples and data points (Sabiiti et al., 2020a).

Measures of bacterial burden, however, often fail to capture the full spectrum of changes in microbial cell physiology induced by sterilising drugs. Cangelosi argues that true treatment efficacy should encompass the cell’s ability to maintain homeostasis and metabolic activity (Cangelosi et al., 1996; Cangelosi and Meschke, 2014). Thus, finding a marker that captures these changes could generate useful information on the true efficacy of antibiotic agents and could help in developing of new antibiotic agents. We have addressed this by creating a molecular assay to expand the scope of the TB MBLA by measuring both pre-16S rRNA in a single RT-qPCR assay.

There is a sharp decrease in pre-16S rRNA expression between days 0 and 3 in all of the patients on treatment which suggests an immediate metabolic response to antibacterial therapy given. To turn these measurements into parameters that could be used to compare treatments we created a mathematical model to calculate the results to measure the rate of maximum pre-16S rRNA synthesis (Rmin) and the time of which corresponds to the time point when 99% of the total decrease is reached (99% of Rmin (T). Notably, higher doses of rifampicin demonstrated increased efficacy, with the 1200 mg dose showing the quickest suppression of RNA synthesis. Rifampicin’s mechanism, targeting DNA-dependent RNA synthesis and inhibiting *de novo* pre-16S rRNA production in susceptible cells, underpins its effectiveness against slow or non-replicating bacteria (Mosaei and Zenkin, 2020).

Our results, although showing a non-significant trend in this small proof of concept study, align with recent findings that suggest that the standard rifampicin doses is suboptimal (Brake et al., 2021). As the dose increased, the value of Rmin and the time to 99% suppression decreased. The maximum tolerated dosage has recently been established at 45mg/kg (Brake et al., 2021). In this study we examine doses much lower than this optimum and this may explain the non-significant trend that we demonstrate on the impact on the pre-16S/16S ratio in a clinical trial setting. A trend that is also found in the clinical efficacy of these sub-maximal doses (Martinecz et al., 2023).

Further insights into these mechanisms were gained by examining our measures in well-charaterised *in-vitro* bacterial growth phases: lag, exponential, and stationary. Our data appears to indicate that the antimicrobial therapy had the effect of moving the expression of ribosomal markers towards a state resembling the stationary phase (a dormancy-like phenotype) marked by a rapid decrease (>98%) in the relative production of pre-16S rRNA. This change was observed when therapy was started, irrespective of the doses. There is a paradox here: rifampicin is most active against slowly replicating cells (Bowness et al., 2015) yet the HIGHRIF 2 regimen is pushing the Mtb cells into a that state difficult to differentiate from stationary-phase or dormancy. Antibiotic treated Mtb organisms are known to adopt a dormancy phenotype with lipid inclusions and phenotypically more resistant to antibiotics with the impact greatest for isoniazid and least for rifampicin (Hammond et al., 2022). This suggests that this effect may be predominantly driven by the isoniazid in this regimen.

Our study confirms recent work by Walters et al., who also explored measures of pre-rRNA abundance as a marker of ongoing metabolic changes during treatment (Walter et al., 2021). Whilst the methodologies differ, the concept of using ribosome synthesis precursors in combination to gauge metabolic activity is similar. The RS ratio method has been shown to be reproducible in a series of animal and human studies (Walter et al., 2021; Dide-Agossou et al., 2022; Cummings et al., 2025; Musisi et al., 2021). The RS ratio shows promise as a mechanism to rank treatment regimens in development (Reichlen et al., 2023). It has also been used in a clinical study with Ugandan and Vietnamese patients highlighting that higher rifampicin doses suppressed ribosome synthesis effectively, underlining the drug’s impact on RNA metabolism or bacterial adaptive responses.

Sequential measurement of ribosomal markers in early phase II studies monotherapy studies will provide an insight into the action of the tested drug (Walter et al., 2021). As anti-tuberculosis therapy is always a combination of compounds, it might also show how combinations combine and might detect potential synergy or antagonism, which is currently not possible. It has been suggested that these patterns may indicate whether a drug has sterilising activity, but these terms that are derived from a *post hoc* interpretation of clinical trial data need further definition. The use of ribosomal marker should make a reassessment of these terms possible.

We have demonstrated the complexity of interpreting culture based data for monitoring treatment response. While TTP serves as a valuable tool for measuring bacterial load, its sensitivity to the metabolic state of TB bacilli necessitates a nuanced approach to data analysis. While the assay offers a semi-quantitative measure of bacterial load by gauging the number of actively respiring bacteria, recent findings from Bowness et al. highlight that TTP is influenced not only by bacterial quantity but also by their metabolic state (Bowness et al., 2015). Identical bacterial concentrations produced longer TTP readings at later stages of TB treatment compared to the early days of therapy, suggesting a shift in bacterial metabolism over time. This provides further data to indicate that bacterial burden measured by different techniques, and metabolic state vary independently. Using the ΔCT and 16S rRNA CT as two independent variables, multiple linear regression enabled us the investigation of the dependence of MGIT TTP on the two independent factors simultaneously. The results clearly showed us that there is an association between MGIT TTP and the two independent variables, ΔCT and 16S rRNA CT, with statistically significant interactions in both cases. We were able to demonstrate, however, that MGIT TTP is more influenced by the physiological and metabolic characteristics *of M. tuberculosis* than by the absolute number of bacteria. This confirms that profound metabolic changes occur in the patient’s pathogen that may not be captured by the culture result. This emphasises that measures of the dormancy phenotype must be considered to fully understand anti-tuberculosis drug activity (Hammond et al., 2015; Lipworth et al., 2016; Hammond et al., 2022). In this study we report a simple multiplex assay that could be applied in clinical trials. This would be facilitate the use of ribosomal ratio monitoring by using a low cost high throughput platform MBLA assay that we describe here (Gillespie and Sabiiti, 2025).

### 4.1 Limitations

As a proof of concept study, the sample size was necessarily small, with the patients being intensively studied. The trends demonstrated provide encouragement that measurement of pre-16S is possible in a single multiplex qPCR in patients recruited in a well-established trial of optimised rifampicin. Measuring relative gene expression by conventional RT-qPCR is inherently cumbersome when the starting RNA concentration is low. A defined detection threshold can help to ensure data accuracy, however, it can lead to a considerable amount of missing data biasing the subsequent data analysis. In our case, it was important to measure the biological changes associated with treatment in the low abundance range that is often masked if high Ct values are excluded from the analysis due to the uncertainty around true amplification. To understand this issue, we performed high output melt-curve analysis in clinical samples with higher than average Ct values and compared the melt-curves to that yielded from highly abundant pre-16S rRNA samples. We found that even in samples with Ct>35 target-specific amplification occurred and therefore we included any data that yielded positive results within 40 PCR cycles. While this reduced the occurrence of missing data, it also meant that one of the criteria of the delta-delta method which states that there is near 100% amplification efficacy of the reference and the target genes have not been always met, which may have limited the robustness of the assay in exchange for making it possible to monitor the very fine metabolic changes when pre-16S rRNA production is heavily and almost unmeasurably suppressed.

### 4.2 Summary

We present a easy to use, low-cost multiplex assay able to measure ribosomal synthesis rapidly that indicates rapid shutdown when bacteria are exposed to chemotherapy. This effect appears to be dose related and could prove useful in rapid assessment of tuberculosis regimens. Since the core TB-MBLA technology is well-established in research practice, it could provide a simple way to build on the expanding evidence of the value or ribosomal ratio to provide insight into tuberculosis chemotherapy design rapidly at low cost that could streamline selection of regimen components.

## Data Availability

The original contributions presented in the study are included in the article/**Supplementary Material**, further inquiries can be directed to the corresponding author/s. The primers analyzed in this study was obtained from VitalBacteriaTM. Requests to access these datasets should be directed to https://www.vitalbacteria.com/.
