# Supplementary material for: Simultaneous measurement of 16S-rRNA and pre-16S-rRNA as a strategy to monitor clinical tuberculosis
Source: Front Antibiot. 2026 Apr 8;5:1760862. doi: 10.3389/frabi.2026.1760862 (PMC13101433; doi:10.3389/frabi.2026.1760862)
Supplement: Supplementary file 1 [file DataSheet1.pdf]

Supplementary material

Extraction control

The purpose of the internal control is to ensure that the extraction efficiency is taken into account. We have added a reference to his process for the benefit of readers.
TB-MBLA uses a novel cell-based extraction control to ensure accurate bacterial load measurement after RNA extraction. The control is added to the sample at a known concentration and amplified alongside M. tuberculosis 16S rRNA in a multiplex RT-qPCR assay. The extraction control's CT value is used to normalize 16S rRNA quantitation, correcting for RNA loss during extraction (e.g., from centrifugation or pipetting).

A serial dilution of the extraction control is prepared and amplified to create a calibration curve linking cell concentration to CT values. According to the TB-MBLA protocol, 100 µl of extraction control (6 log10 CFU/ml) is spiked into 900–1000 µl of the sample. If RNA extraction is 100% efficient, the total RNA yield from the control equals that from 5 log10 CFU/ml cells, giving a CT of ~23.71.

Any deviation from this value is corrected using:
CT(norm., target) = CT(target) − (CT(EC test) − CT(EC calibrator))

After normalization, bacterial load is calculated using:
concentration = 10(−0.290×CT + 10.561)

Multiplex RT-qPCR development

Pre-16S rRNA and 16S rRNA fragments of the *rrn* operon of Mtb were identified using the NCBI nucleotide database. Primer specificity was tested *in silico* using the online Primer-BLAST tool on the NCBI website (https://www.ncbi.nlm.nih.gov/tools/primer-blast/). The *M. tuberculosis* H37Rv laboratory strain was used as a PCR template. Primer parameters and exon/intron selection were kept as default settings. To assess primer specificity against other Mycobacteria species, the database parameters and organism section were modified to include Refseq representative genomes of Mycobacteria spp. Primers and probes were tested for potential primer dimer formation using the online Oligo Analyzer tool on the Integrated DNA Technologies website (https://eu.idtdna.com/pages). Pre-16S rRNA primers were designed to target a region approximately 220 base pairs upstream of the M. tuberculosis 16S rRNA gene coding sequence. This spacer element is cleaved from the primary rRNA transcript during maturation by RNAse III enzyme activity. The 16S rRNA primers, added to the multiplex PCR mix, amplify an approximately 70 base pair region of the 16S rRNA gene.

Supplementary Table 1 Delta Ct values derived from raw Rt-qPCR Cts across a conventional growth curve. Average delta CTs were calculated to illustrate the correlation between metabolic activity and the various stages of bacterial growth Low delta Ct during the logarithmic phase indicates heightened ribosome synthesis associated with active replication which diminishes as bacteria reaches static

phase. Medium delta Ct measured in early days of growth suggests the adaptation to new environment typical of the lag phase.

	C_T pre-16S rRNA	C_T 16S rRNA	Delta C_T	Replication state and median
Day 2	25.81	21.77	4.41	**LAG** ΔC_T=4.41
Day 5	22.15	20.35	1.94	**LOG** ΔC_T=2.77
Day 7	20.9	17.18	2.77	
Day 16	18.67	13.95	4.93	
Day 20	15.85	9.49	6.24	**STATIONARY** ΔC_T=6.61
Day 35	19.6	12.63	6.98	

Supplementary Table 2

**Parameter**	**Value**	**95% CI**	**P-value**
$m_{CT\,16S}$	0.5494	0.121 to 0.9778	0.0125
$m_{\Delta CT}$	1.089	0.5606 to 1.617	<0.0001
b(intercept)	−4.837	−12.01 to 2.339	0.1839

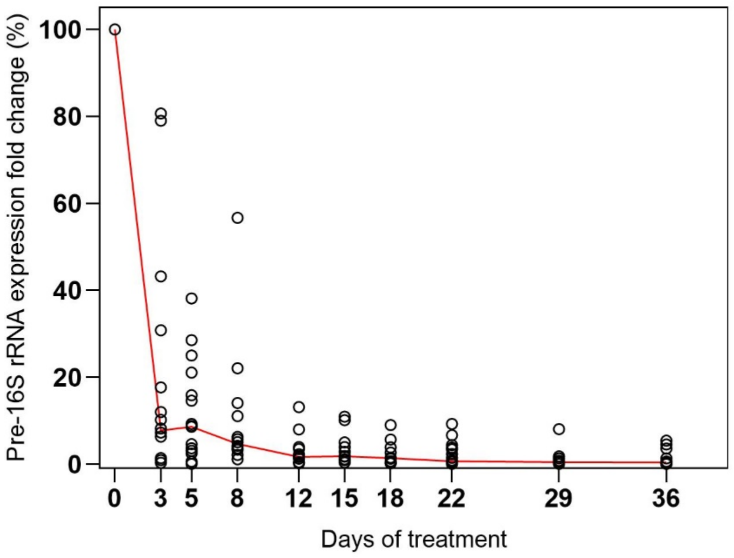

Supplementary Figure 1 Pre-16S rRNA fold change measured during HRZE therapy, including standard (600 mg) or higher doses of rifampicin (900 mg or 1200 mg). The analysis incorporated 163 data points from the aggregated patient dataset, with curves fitted to median values. The rapid decline in pre-16S rRNA expression observed between days 0 and 3 indicates an immediate metabolic response to antibacterial therapy.

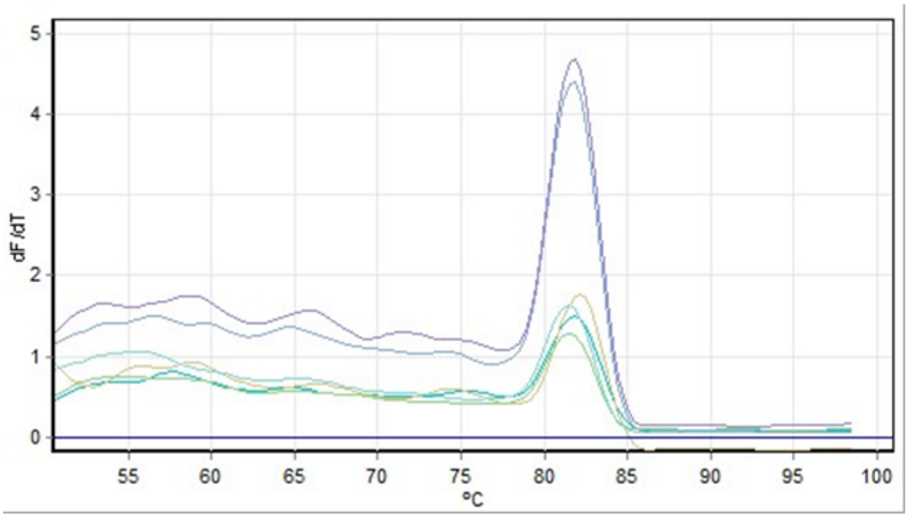

No.	Color	Name	Pre-16S Ct	Peak 1	Peak 2	Peak 3	Peak 4
11		P34 V15 neat MM2	38.3	53.8	57.7	64.7	75.3
12		P34 V15 neat MM2		55.7	58.8	66.3	74.5
13		P34 V13 1/10 MM2	28.5	53.8	55.3	65.0	81.5
14		P34 V13 1/10 MM2		55.5	65.0	81.5	86.5
15		P82 V1 1/10 MM2	20.6	56.5	59.5	64.5	74.0
16		P82 V1 1/10 MM2		53.5	58.7	65.8	71.5

Supplementary Figure X Analysis of melt-curves for low abundant targets
